# Health professional education in autism and intellectual disability: systematic review

**DOI:** 10.1192/bjo.2025.10842

**Published:** 2025-10-10

**Authors:** Catherine Franklin, Sinead Green, Katie Brooker, Ruby de Greef, Carla Meurk, Edward Heffernan

**Affiliations:** School of Public Health, Faculty of Health, Medicine and Behavioural Science, The University of Queensland, Herston, Queensland, Australia; Queensland Centre of Excellence in Intellectual Disability and Autism Health, Mater Research Institute-UQ, The University of Queensland, Brisbane, Queensland, Australia; Forensic Mental Health Group, Queensland Centre for Mental Health Research, West Moreton Hospital and Health Service, Wacol, Queensland, Australia

**Keywords:** Intellectual disability, autism, health, mental health, health professional education

## Abstract

**Background:**

Health and mental health professionals often lack knowledge and confidence to provide quality healthcare to people with intellectual disability and those on the autism spectrum. Educational interventions are proposed as solutions, but their effectiveness and optimal characteristics remain unclear.

**Aims:**

To evaluate the effectiveness of educational interventions in improving health professionals’ knowledge, skills, attitudes, confidence and/or self-efficacy in providing care to people with intellectual disability and those on the autism spectrum.

**Method:**

A mixed-methods systematic review was conducted searching six major databases, adhering to PRISMA guidelines (PROSPERO CRD42022309194). Studies were included if they assessed outcomes of educational interventions aimed at improving health professionals’ capacity to provide care to people with intellectual disability and/or those on the autism spectrum.

**Results:**

We identified 34 studies: five focused on intellectual disability, two on intellectual and developmental disabilities, and 27 on autism. All reported positive findings, although heterogeneity of measures limited synthesis. Most studies (30 out of 34) employed single group pre-test/post-test designs, with only nine using validated outcome measures. Only eight studies reported co-design or co-delivery involving people with lived experience.

**Conclusions:**

Educational interventions demonstrate positive effects on heath professionals’ capacity to provide care. Significant gaps include limited evidence for adult-focused interventions, uncertainty about optimal delivery modes and duration, and minimal inclusion of people with lived experience in intervention design and delivery. Future interventions should involve people with lived experience in design and delivery, and incorporate validated outcome measures to enhance evidence quality.

Health and mental health professionals lack knowledge and confidence to provide healthcare to individuals with intellectual and developmental disabilities^1^ and autistic individuals.^2^ Negative attitudes have also been reported, contributing to stigma.^3^ This contributes to poorer health outcomes, a finding that has been highlighted as contributing to neglect and inadequate treatment in inquiries in Australia^4^ and England,^5^ prompting recommendations for education at undergraduate, postgraduate levels and in the workplace for practising professionals.^6^ England has introduced legislation to mandate this training for all health and social care staff.^7^ Education in this area needs to increase knowledge about the nature of intellectual disability and autism, as well as to understand some of the diagnostic and management issues that are important in providing healthcare, including diagnostic overshadowing.^8^

However, the evidence for such education to change knowledge, attitudes and confidence is unclear. Some recent systematic reviews have investigated some aspects of this question, including a systematic review of post-graduate medical education in training programmes,^9^ and a systematic review of outcomes of autism-related training for physicians.^10^ Whether there is a minimum dose (either in duration or frequency) of education required to maintain improved knowledge and attitudes is also unclear, but of relevance to employers, as this education will need to be conducted in paid work hours. In recent years, there have also been calls for improved standards of evaluation of education, including use of standardised outcome measures and improved quality of study design.^11^ The inclusion of individuals with intellectual disability and autistic individuals is valued and recommended in research in this area, and embedding these principles in research design and delivery is an emerging focus in the literature.^12^

The objective of this review was to evaluate the evidence base for the use of educational interventions to build the capacity of health professionals to work more effectively with individuals with intellectual or developmental disabilities, including autistic individuals. This included answering the following questions:

- Is education effective in improving knowledge, attitudes, confidence and/ or self-efficacy of healthcare professionals to provide healthcare to individuals with intellectual or developmental disabilities?
- What is the frequency and nature of the involvement of individuals with intellectual or developmental disabilities, including autistic individuals?
- Are there particular modes, or duration of education that are more effective?
- What outcome measures are used to evaluate effectiveness of education in this area?

## Method

Our systematic review is reported using the Preferred Reporting Items for Systematic Reviews and Meta-Analyses (PRISMA) Statement,^13^ and the corresponding checklist can be viewed in Supplementary Table 1 available at https://doi.org/10.1192/bjo.2025.10842. The protocol was prospectively registered with PROSPERO (CRD42022309194) on 24 March 2022.^14^

### Eligibility criteria

To be included, studies had to report quantitative and/or qualitative outcomes of educational interventions aimed at building the capacity of health professionals to work effectively with individuals with intellectual or developmental disabilities (including autistic individuals) of any age. Outcomes included clinician knowledge, attitudes, confidence (a broad term in earlier research) and/or self-efficacy (context-specific confidence). The focus was on qualified health professionals (e.g. medical, nursing, allied health), not undergraduate students; studies with mixed cohorts required >50% healthcare professionals to be eligible. This distinction reflects differing educational needs and motivations between practising clinicians and students. Eligible interventions used any method or mode of training, but needed a clear goal of enhancing clinical care capacity not solely diagnostic- or treatment-specific training.

Studies were included if they met all the following:

- participants were health professionals working in mainstream (non-specialist intellectual disability, intellectual and developmental disabilities, or autism health) settings, including community and hospital settings;
- interventions aimed to build clinician capacity to work effectively with individuals with intellectual or developmental disabilities (including autistic individuals) and included an educational component;
- quantitative and/or qualitative within or between-individual comparisons were reported;
- outcomes included at least one of the following: clinician knowledge, attitudes, confidence or self-efficacy.

Studies were excluded if they met one or more of the following:

- >50% participants were students, trainees (including professionals in training) or non-health professionals (e.g. disability support workers);
- interventions focused solely on diagnosis or screening, (e.g. diagnosis of foetal alcohol spectrum disorder, conducting cervical screening);
- no relevant quantitative or qualitative findings were reported (e.g. studies reporting only diagnostic outcomes without assessing health professional knowledge, attitudes, confidence or self-efficacy).

### Search strategy

Studies were identified by searching PubMed, PsycINFO, ProQuest, Web of Science, CINAHL, EMBASE and the grey literature. The Cochrane Library and PROSPERO database were also searched to ensure no other systematic reviews were planned or had been published in this area. A University of Queensland librarian reviewed the search strategy. The search was not restricted to date, thus a variety of search terms that included historical terms for intellectual disability and autism were included. Broad terms for clinicians were used to cover the wide variety of health professionals, and possibility that interventions were only conducted on one professional group. Search terms are summarised in Table 1.

**Table 1:** Search terms

Participants	Intervention	Population	Outcomes
‘staff’ OR ‘health* work*’ OR ‘clinician*’ OR ‘health professional*’ OR ‘health practitioner*’ OR ‘nurs*’ OR ‘psych*’ OR ‘social worker*’ OR ‘occupational therapist*’ OR ‘paramedic’ OR ‘gp’ OR ‘general practitioner*’ OR ‘family medicine practitioner*’ OR ‘paediatric*’ OR ‘physician*’ OR ‘doctor*’ OR ‘resident* OR ‘intern*’	strateg* OR intervention* OR model* OR education OR train* OR ‘medical education’	‘intellectual disabilit*’ OR ‘Complex communication need*’ OR ‘Developmental disabilit*’ OR ‘Intellectual handicap*’ OR ‘Intellectual impairment*’ OR ‘Learning disabilit*’ OR ‘Mental handicap*’ OR ‘Mental retardation’ OR autis* OR ‘asperger*’	capacity OR knowledge OR awareness OR attitude* OR ‘skill*’

Each search term was limited to title/abstract, each search string was connected using the Boolean operator ‘AND’, and the * indicates that the search is broadened to include any words starting with these letters preceding the *.

### Study selection

Authors C.F. and K.B. undertook dual independent blinded reviews of the titles and abstracts of articles remaining after removal of duplicates, using the Covidence platform on Windows and macOS (Veritas Health Innovation, Melbourne, Australia; see https://www.covidence.org). C.F. and K.B. then undertook dual independent blinded review of the full text of included articles. Further information was sought from authors of two included publications to clarify published information relevant to inclusion. Each article described a unique study, and there were no studies that were reported in more than one article. Disagreements between reviewers were resolved at each stage through discussion, and consensus was able to be reached without involvement of a third reviewer.

### Data collection, extraction, synthesis and analysis

Data was extracted on the Covidence platform, using a form designed for this study by C.F. Data extraction was dually independently undertaken by C.F. and S.G. Data extraction commenced on 31 August 2024 and was completed on 17 December 2024. Conflicts were resolved through discussion and consensus achieved at each stage of the above process.

Data items that were extracted for each study were: reference details; country (where intervention was delivered); consumer involvement (co-design (yes/no) and co-delivery (yes/no)); delivery mode (face to face, online, video, written); format (didactic, interactive, mixed (noting if Extension for Community Healthcare Outcomes (ECHO)); duration of intervention; setting (primary care, community health, community mental health, paediatric hospital, mental health in-patient care); study design (qualitative, single group pre-test/post-test, prospective waitlist control, non-randomised two-group, partial stepped wedge randomised controlled trial); outcome measure area (knowledge, attitudes, confidence, self-efficacy, skills, behaviour) and name of measure; quantitative methods; and results. For studies that included qualitative methods, the aim, approach and main themes were summarised. Missing data were denoted by ‘NS’ (not stated). Each data point was extracted by two independent reviewers, C.F. and S.G. Any differences in data were discussed, and consensus was reached.

Participant numbers were collected, along with their professional group (medical, nursing, allied health professionals – specific professional listed if identifiable). The number of participants who completed the intervention and post-intervention evaluation were recorded, compared with those who commenced and provided baseline data. The objective of the intervention was recorded, in addition to the area of focus (intellectual disability, intellectual and developmental disabilities or autism), whether it focused on paediatric or adult healthcare, and any specific education focus (the diagnosis of autism or intellectual disability, general clinical care).

Evaluation methods were a focus of this review. Data was collected to describe the study design, outcome measures and data points and results, in those studies where published or validated measures were used. In those studies where qualitative methods of evaluation were reported, the framework, analytic process and focus of investigation and themes were recorded for each study.

### Study risk-of-bias assessment

The variation in methods of assessment and evaluation of health professional education made it difficult to meaningfully compare the quality and risk of bias of included studies. Therefore, the Mixed-Methods Appraisal Tool (MMAT)^15^ was used to assess qualitative methods, and the Medical Education Research Study Quality Instrument (MERSQI) was used to assess quality of quantitative studies.^16^ The MMAT is a critical appraisal checklist of the methods used.^15^ The MERSQI, on the other hand, is quantitatively focused and has a scoring system, but it does not specify specific thresholds of methodological quality. It has been validated as a reliable tool for this purpose.^11^ The MERSQI consists of ten items, with total possible scores ranging from 5 to 18. One study suggested using a score of 14 to denote high-quality studies,^17^ and another agreed and further proposed suggested ranges of 5–9 for low-quality studies and 9–14 for medium-quality studies.^10^ Authors C.F. and S.G. undertook dual blinded independent assessments, applying the MERSQI and the MMAT (where appropriate), using these thresholds. Disagreement in quality ratings were resolved through discussion between the two reviewers, until consensus was reached. Risk of bias in data extraction was minimised by using two independent blinded investigators (C.F. and S.G.) to assess each study via the Covidence platform. Disagreements in risk of bias assessments were resolved through discussion.

Selective reporting bias was assessed by comparing planned and reported outcome measures for each study via checking published trial protocols and, where not published, the methods specified in the publications.

### Effect measures

*P*-values were selected to indicate statistical significance across studies, as they were commonly reported in this study and allow some comparison between studies.

### Synthesis methods

Synthesis of mixed methods studies was initially conducted by analysing quantitative and qualitative findings separately.^18^ Data was extracted and recorded using the Covidence platform, and then collated into a table to record characteristics of individual study design (Tables 2 and 3). Qualitative findings were then collated in a separate table that included data on methods, themes and findings (Table 4). The frequency of each data field in Tables 2 and 3 were collated in Table 9 to synthesise findings.

**Table 2:** Intellectual and developmental disabilities study design characteristics

Study identifier	Country	Study						Study design	Outcome measures	
		Co-design	Co-delivery	Delivery mode	Format	Duration	Setting		Domain	Measure
**Intellectual disability – clinical care of adults**										
Dagnan 2018^19^	UK	No	No	Face to face	Interactive	10 or 20 h	Community mental healthcare	SGPP, Qualitative (post)	Attitudes, Confidence, Self-efficacy	ATTID, TCS-ID, GSE
Eagleson 2022^20^	Australia	Yes	No	Online	Didactic	Not stated	Mental healthcare	SGPP	Knowledge, Attitudes, Confidence, Skills	Bespoke
Harper 1992^21^	USA	No	No	Video, Written	Didactic	45 min	Health service providers	SGPP	Knowledge	Bespoke
Melville 2006^22^	UK	No	Yes	Written, Written + Face to face	Mixed	45 min (Written), 3 h (Face to face)	Primary care	Non-randomised, two group	Knowledge, Self-efficacy	Bespoke
Bessell 2023^23^	Australia	No	No	Online	Mixed (ECHO)	15 h	Primary care, Community mental healthcare	Prospective waitlist control	Knowledge, Confidence	Bespoke
**Intellectual and developmental disabilities – clinical care of children**										
McConkey 2014^24^	Macedonia	Yes	No	Face to face	Interactive	20 h	Community healthcare	SGPP	Knowledge, Atittudes, Confidence	Bespoke
Sadoo 2022^25^	Uganda	No	No	Face to face	Interactive	2 days	Community healthcare	SGPP Qualitative	Knowledge, Confidence	Bespoke

SGPP, single-group pre-test/post-test; ATTID, Attitudes to the Treatment of People with Intellectual Disabilities in Mainstream Services; TCS-ID, Therapy Confidence Scale–Intellectual Disabilities; GSE, General Self-Efficacy Scale; ECHO, Extension for Community Healthcare Outcomes. Study identifier: only the first author is noted, with year of publication. Bespoke refers to unpublished measures, constructed for purpose by authors.

**Table 3:** Autism study design characteristics

Study identifier	Country	Study						Study design	Outcome measures	
		Co-design	Co-delivery	Delivery mode	Format	Duration	Setting		Domain	Measure
**Autism – screening in children**										
Balogh 2015^26^	Canada	No	No	Face to face, Online	Mixed	2 days + Online	Primary care	SGPP	Knowledge, Behaviours	Bespoke
Bordini 2015^27^	Brazil	No	No	Face to face	Didactic	15 h	Community healthcare, Primary Care	SGPP	Knowledge, Behaviours	Bespoke, Referrals
Carbone 2016^28^	USA	No	Yes^a^	Face to face	Interactive	Not reported	Community healthcare	SGPP	Behaviours, Self-efficacy	Number of screenings, Bespoke
Johnson 2012^29^	USA	No	No	Online	Mixed	1 h	Paediatric hospital	SGPP	Not stated	Bespoke
Lucarelli 2018^30^	USA	No	No	Face to face, Online	Didactic	Not reported	Hospital (emergency)	SGPP	Knowledge, Attitudes, Confidence	Bespoke
Mahoney 2023^31^	USA	No	No	Face to face, Online	Interactive	6 h	In-patient healthcare	SGPP	Knowledge, Confidence, Other	Bespoke
Mazurek 2018^32^	USA	No	Yes	Face to face, Online	Mixed (ECHO)	9 h	Community healthcare, Primary care	SGPP, Qualitative	Self-efficacy, Behaviours	PCASE, Number of screenings
Swanson 2014^33^	USA	No	No	Face to face	Interactive	2 days	Community healthcare	SGPP	Behaviours	Bespoke
van’t Hof 2021^34^	Netherlands	No	No	Online	Didactic	4.5 h	Community healthcare	SGPP	Knowledge, Attitudes, Confidence, Satisfaction	AKQ-P, CAMI, Bespoke
**Autism – clinical care of children**										
Ashburner 2015^35^	Australia	No	No	Face to face	Other	>3 days	Community healthcare	SGPP, Qualitative	Knowledge, Confidence	Bespoke
Bellesheim 2020^36^	USA	No	Yes^a^	Online	Mixed (ECHO)	9 h	Community healthcare	SGPP	Behaviours	Bespoke
Donnelly 2023^37^	USA	No	No	Online	Interactive	90 min	Community healthcare, In-patient care	SGPP	Knowledge, Attitudes, Confidence	Bespoke, EBPAS, CCPAI, CHABA
Eray 2017^38^	Turkey	No	No	Face to face	Didactic	2 h	Primary care	SGPP	Knowledge	Bespoke
Silva 2018^39^	Brazil	No	No	Face to face, Online	Didactic	4 h	Hospital mental healthcare	SGPP	Knowledge, Satisfaction, Attitudes	KAP, Bespoke
Gore 2024^40^	Australia	No	No	Face to face, Online	Mixed	Not reported	Community healthcare	SGPP	Knowledge, Self-efficacy	Bespoke
Jonsdottir 2020^41^	Iceland	No	No	Face to face	Didactic	4 h	Community healthcare	Retrospective pre-post, no control group	Knowledge, Confidence	Bespoke
Ong 2021^42^	Australia	Yes	No	Face to face	Didactic	2 h	In-patient healthcare	SGPP	Knowledge, Confidence	Bespoke
Pasco 2014^43^	Romania	No	No	Face to face, Online	Mixed	Varying	Community healthcare	SGPP	Knowledge	Bespoke
Sengupta 2022^44^	India	No	Yes	Online	Mixed (ECHO)	26 h	Community healthcare, Hospital healthcare	SGPP, Qualitative	Knowledge, Satisfaction, Self-efficacy	Bespoke, PCASE
**Autism – clinical care of adults**										
Ben-Sasson 2018^45^	Israel	No	No	Face to face	Interactive	2 days	Community healthcare	SGPP	Knowledge, Self-efficacy, Satisfaction	ASD-KS, Bespoke
Dreiling 2022^46^	USA	No	No	Online	Mixed (ECHO)	15 h	Communitymental healthcare	SGPP	Knowledge, Satisfaction, Self-efficacy	Bespoke, PCASE
Giachetto 2019^47^	Uruguay	No	No	Online	Didactic	20 h	Community healthcare	SGPP	Knowledge	Bespoke
Giarelli 2012^48^	USA	No	No	Face to face	Interactive	2 days	In-patient healthcare	SGPP	Knowledge	Bespoke
Malow 2023^49^	USA	Yes	Yes	Online	Mixed (ECHO)	12 h	Community healthcare, Primary care	SGPP	Knowledge, Self-efficacy	Bespoke
Mazurek 2020^50^	USA	No	Yes^a^	Online	Mixed (ECHO)	24 h	Community healthcare, Primary care	Partial stepped wedge RCT	Knowledge, Self-efficacy	Bespoke
Mazurek 2020^51^	USA	No	Yes	Online	Mixed (ECHO)	12 h	Community healthcare	SGPP	Knowledge, Self-efficacy	Bespoke, PCASE
McGonigle 2014^52^	USA	Yes	No	Online	Didactic	1.5–3 h	Hospital care (emergency)	SGPP	Knowledge, Confidence	Bespoke

SGPP, single-group pre-test/post-test; ECHO, Extension for Community Healthcare Outcomes; PCASE, Adapted, shortened version of the Primary Care Autism Self-Efficacy survey; AKQ-P, Autism Spectrum Disorder Knowledge Questionnaire – Physician Edition; CAMI, Dutch translation of the Community Attitudes to Mental Illness questionnaire; EBPAS, Evidence-Based Practice Attitude Scale; CCPAI, Confidence in Coping with Patient Aggression Instrument; CHABA, Challenging Behaviour Attributions Scale Short Form; KAP, Knowledge, Attitudes, Practice; ASD-KS, Autism Knowledge and Self-Efficacy Questionnaire. a. Delivered by a parent of an autistic child, no involvement by autistic person themselves. Study identifier: only the first author is noted, with year of publication. Bespoke refers to unpublished measures, constructed for purpose by authors.

**Table 4:** Qualitative design and outcome of mixed-method studies

Study identifier	Qualitative aims	Approach	Method	Analysis process	Areas for investigation	Themes
**Intellectual disability – clinical care of adults**						
Dagnan 2018^19^	To add to the understanding of the effect of training and to gain insight from therapists’ reflections on the impact of training on their clinical practice	Thematic analysis	Individual Interviews, two questions	No coding method described. Themes described and collated, then validated by a second rater	Whether skills acquired in training were used and how they were used	Increased awareness and sensitivity Adaptation and simplification of materials Adaptation and simplification of communications Adapting interventions
**Intellectual and developmental disabilities – clinical care of children**						
Sadoo 2022^25^	To explore perceptions of the training programme and impact on confidence levels, attitudes and practice	Thematic framework approach	Focus group discussions using semi-structured interview guide	Coding framework, descriptive analysis described	Central beliefs of the psychologists regarding the contribution of nurses/social workers to the diagnostic process in both research conditions	Training was beneficial Resultant improved communication skills positively influenced relationships with caregivers Intervention changed attitudes and behaviour because of clearer understanding of causes of disability and improved knowledge of diagnosis and management
**Autism – screening in children**						
Mazurek 2018^32^	To evaluate the feasibility of the model and to examine the effects of this training approach on primary care provider self-efficacy and practice change	None stated	Qualitative written survey questions	None stated	Perceptions of changes in practice, changes in relationships and interactions with patients and autism and their families, and potential impact on their communities	Enhanced screening and evaluation Increased use and knowledge of autism resources Improved access to care for families Greater autism knowledge Increased local expertise Improved relationships with families
**Autism – clinical care of children**						
Ashburner 2015^35^	To evaluate workplace training	None stated	Qualitative written survey questions	Two coders used an inductive process to search for preliminary and higher order themes in responses. Responses were grouped and analysed statistically	Successes, challenges, pairing preferences, and recommended improvements	Successes in co-mentoring Challenges in co-mentoring Pairing preferences for co-mentoring Recommendations for improving co-mentorship programme
Sengupta 2022^44^	To evaluate the relevance and effectiveness of an evidence-based tele-mentoring model Extension for Community Healthcare Outcomes (ECHO) Autism in increasing paediatricians’ access to best-practice care for children with autism spectrum disorder in low- and middle-income country contexts	Thematic analysis hybrid of approaches (deductive *a priori* coding and data-driven inductive approach)	Qualitative written survey questions	Coding manual was developed. Codes were used to identify themes. One coder and one reviewer	Relevance to learner, benefits of participation, changes to behaviour as result of training	Case-based discussion format was much more helpful than theory Layering of concepts across sessions helped learning Parent of autistic child as member of expert hub was very valuable

Study identifier: only the first author is noted, with year of publication.

## Results

### Study selection

**Fig. 1:**
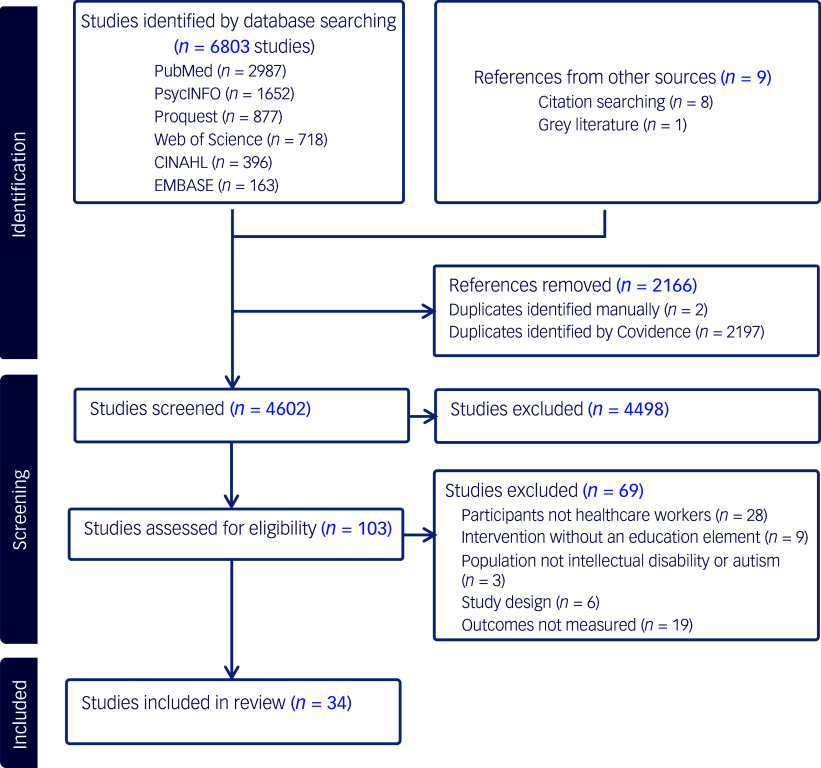
PRISMA flowchart for study identification, screening and inclusion.^13^

A total of 6803 studies were identified on searching relevant databases. After duplicates were removed, 4602 studies remained for screening of title and abstract; 4498 of these did not meet inclusion criteria and were removed. After assessment for eligibility, another 69 studies were excluded. The main reasons for exclusion were that the participant group did not consist of predominantly health professionals (*n* = 28) or that there was no reporting of outcome measures or methods (*n* = 19). This left 34 studies that met the eligibility criteria for the review (Fig. 1).

### Study characteristics

The design characteristics of the 34 studies are summarised in Tables 2 and 3, where they are arranged according to the specific focus of the education (intellectual disability, intellectual and developmental disabilities, or autism). All studies included quantitative measures, no studies were purely qualitative, and five of the 34 studies used mixed methods. Table 4 summarises the qualitative design of those studies where qualitative analysis was reported.

The earliest study was published in 1992, although the vast majority were published between 2014 and 2023. In four studies, the focus of the educational intervention was intellectual disability clinical care in adults.^19–23^ There were two intellectual and developmental disabilities educational interventions focused on clinical care in children.^24,25^ The remaining 27 studies were autism educational interventions: 19 focused on autism in children and eight focused on autism in adults. Of the 19 studies that focused on autism in children, nine focused on screening and diagnosis of autism^26–34^ and ten focused on clinical care.^35–44^ The eight educational interventions that covered autism in adults all focused on clinical care.^45–52^

### Risk of bias in studies

The MERSQI was used to assess quality of quantitative data, with scores presented in; Table 5. Only two studies were rated high quality, 22 studies were rated moderate and ten were rated low. Notably, just 11 of the 34 studies used validated, published scales; the remainder relied on unvalidated, bespoke measures. The MMAT was applied to mixed-methods studies, with the lowest scoring domain determining overall quality. Two studies achieved 80% (four stars) and three scored 60% (three stars). A common limitation was the lack of rationale for using mixed methods. Higher-scoring studies reported a clear qualitative framework and methods, whereas lower-scoring ones did not.

**Table 5:** Quality assessment

Study identifier	Quality assessment			
	MERSQI (/15)	MMAT (%)		
		Qualitative	Quantitative	Mixed
**Intellectual disability – clinical care of adults**				
Dagnan 2018^19^	10	100	100	80
Eagleson 2022^20^	9			
Harper 1992^21^	10			
Melville 2006^22^	10.5			
**Intellectual and developmental disabilities – clinical care of children**				
Bessell 2023^23^	10			
McConkey 2014^24^	8.5			
Sadoo 2022^25^	14.5	100	80	80
**Autism – screening in children**				
Balogh 2015^26^	12.5			
Bordini 2015^27^	11.5			
Carbone 2016^28^	11.5			
Johnson 2012^29^	5.5			
Lucarelli 2018^30^	10			
Mahoney 2023^31^	9.5			
Mazurek 2018^32^	9.5	60	80	60
Swanson 2014^33^	10.5			
van’t Hof 2021^34^	14.5			
**Autism – clinical care of children**				
Ashburner 2015^35^	10	80	60	60
Bellesheim 2020^36^	9.5			
Donnelly 2020^37^	13			
Eray 2017^38^	7			
Silva 2018^39^	10			
Gore 2024^40^	9			
Jonsdottir 2020^41^	10			
Ong 2021^42^	6.5			
Pascoe 2014^43^	4.5			
Sengupta 2022^44^	10.5	100	80	80
**Autism – clinical care of adults**				
Ben-Sasson 2018^45^	10			
Dreiling 2022^46^	10			
Giachetto 2019^47^	8.5			
Giarelli 2012^48^	6.5			
Malow 2023^49^	11			
Mazurek 2020^50^	10			
Mazurek 2020^51^	12			
McGonigle 2014^52^	7.5			

MERSQI, Medical Education Research Study Quality Instrument; MMAT, Mixed-Methods Appraisal Tool. Study identifier: only the first author is noted, with year of publication.

### Results of individual studies

The results of individual studies are represented in Tables 4 (qualitative), 6 (knowledge and attitudes), 7 (confidence and self-efficacy) and 8 (behaviour). Three studies reported item-level statistical analysis but not of total scores.^21,29,43^ Three different studies reported scores, but did not perform statistical analyses.^33,47,48^ All 29 studies that reported statistical analyses reported statistically significant findings in at least one outcome area. Findings were similarly positive across each subject area of intellectual disability, intellectual and developmental disabilities, and autism. Qualitative data from the five mixed-methods studies also demonstrated positive findings and tended to focus on the effects of the training on clinical care (Table 4). Only the mixed-methods study by Sengupta et al^44^ described participant feedback relating to the inclusion of people with lived experience in the delivery of the education, noting that participants found it very valuable (Table 4).

### Results of syntheses

Table 9 shows that only eight out of 34 studies reported co-design or co-delivery, with just one incorporating both. Of the six co-delivered studies, three involved individuals with intellectual disability or autistic individuals, and three involved parents of autistic children. **I**nterventions were delivered across 15 countries – 26 in high-income countries (including 15 in the USA, five in Australia, two in the UK) and eight in low- and middle-income countries. Delivery modes varied: 13 were fully online, 12 were face to face, seven used both and two combined written with either video or face-to-face formats. Twelve studies delivered education of 11–20 h, whereas nine were 1–5 h with positive findings. Most interventions were workplace-based, primarily in community health settings.

Most studies (30 out of 34) used a single-group pre-test/post-test design, with 15 collecting data only immediately pre- and post- intervention. Five studies included a qualitative component, although only three of these reported a guiding framework – all using thematic analysis. Because of varied outcome measures, effectiveness by delivery mode (online versus face to face) could not be reliably compared. However, all studies using statistical analyses reported at least one statistically significant positive finding in knowledge, attitudes, confidence and/or self-efficacy. Of the six studies assessing behaviour, five used objective measures, such as the number of cases referred for assessment or use of autism screening measures. Data were typically collected immediately pre- and post- intervention; just over half included a 3-month follow-up, with some extending further to assess for sustained impact. Knowledge was the most assessed outcome, followed by confidence, self-efficacy and attitudes. Behaviour was the least frequent outcome measured, but had some of the longest follow-up intervals, including 6 months, 12 months and 4 years (Tables 8 and 9). Across all studies, 15 studies only assessed for effects immediately following the education, 11 studies for up to 6 months and 2 studies for over 12 months (Table 9).

**Table 6:** Knowledge and attitudes results

Study identifier		Participants		Knowledge										
		Completed	Profession	Scale	Pre		Post		3 months		6 months		12 months	
					Mean	s.d.	Mean	s.d.	Mean	s.d.	Mean	s.d.	Mean	s.d.
**Intellectual disability – clinical care of adults**														
Dagnan 2018^19^		42/68	Allied health											
	PWP													
	HIP													
Eagleson 2022^20^		60/351	Medical, Nursing, Allied health											
Harper 1992^21^		31/Not reported	Medical	Bespoke	85	Not reported	86	Not reported						
			Medical student		87	Not reported	88	Not reported						
			Nursing		80	Not reported	83	Not reported						
Melville 2006^22^		63/79	Nursing	Bespoke										
	Written				32.9	Not reported			39.6**	Not reported				
	Written + Face to face				32.4	Not reported			35.6**	Not reported				
	Control				31.5	Not reported			33.4	Not reported				
Bessell 2023^23^		62/101	Medical, Nursing, Allied health	Bespoke										
	Intervention				2.8	0.4	3.6	0.5						
	Control				2.9	0.7	2.9	0.6						
**Intellectual and developmental disabilities – clinical care of children**														
McConkey 2014^24^		19/19	Nursing	Bespoke	57.0	Not reported	84***	Not reported						
Sadoo 2022^25^		64/93	Medical, Nursing	Bespoke	4.0	Not reported					7.0	Not reported		
**Autism – screening in children**														
Balogh 2015^26^		26/Not reported	Medical, Nursing	Bespoke										
	Intervention				2.6	0.7					3.1	0,6		
	Control				2.6	1.1					2.3	1.1		
Bordini 2015^27^		22/29	Medical (Paediatrics, General practice)	Bespoke	6.7	Not reported	9.2**	Not reported						
Carbone 2016^28^		43/43	Medical (General practice)											
Johnson 2012^29^		599/604	Nursing	Bespoke	5.5	Not reported							8.7^a^	Not reported^a^
Lucarelli 2018^30^		54/129	Nursing, Allied health	Bespoke	3.3	1.0	4.3***	1.0						
Mahoney 2023^31^		107/300	Nursing	Bespoke	4.2	0.8							4.3	0.7
Mazurek 2018^32^		18/26	Medical (General practice), Nursing											
Swanson 2014^33^		118/Not reported	Medical (Paediatrics)											
van’t Hof 2021^34^		78/93	Medical	AKQ-P										
				General	7.2	1.3	7.8**	1.0	7.3	1.2				
				Specific	5.9	1.6	7.1**	1.4	6.5	1.7				
**Autism – clinical care of children**														
Ashburner 2015^35^		32/Not reported	Allied health (Occupational therapy)	Bespoke	16.9	4.8	21.9***	2.6						
Bellesheim 2020^36^		28/Not reported	Medical											
Donnelly 2020^37^		233/308	Medical (Paediatrics), Nursing, Allied health	Bespoke	9.5	1.4	10.2***	1.1						
Eray 2017^38^		75/79	Medical (General practice)	Bespoke	34.7	Not reported	88.0***	Not reported						
Silva 2018^39^		14/14	Nursing, Allied health	KAP	53.5	12.0	65.5**	8.3						
Gore 2024^40^		38/344	Nursing	Bespoke	35.9	3.3	36.9	2.8						
Jonsdottir 2020^41^		56/56	Medical, Nursing	Bespoke	2.1	0.5	3.1***	0.3						
Ong 2021^42^		8/12	Allied health	Bespoke	3.3	0.6	3.8*	0.6						
Pasco 2014^43^		329/588	Medical, Nursing, Allied health	Bespoke	17.5	Not reported	20.5	Not reported						
Sengupta 2022^44^		62/88	Medical (Paediatrics)	Bespoke	63.2	Not reported	78.9***	Not reported						
**Autism – clinical care of adults**														
Ben-Sasson 2018^45^		26/Not reported	Allied health (Physiotherapy)	ASD KSEQ	4.1	0.8	4.7	0.5						
Dreiling 2022^46^		51/86	Allied health	Bespoke	11.1	2.8					14.3***	2.6		
Giachetto 2019^47^		38/Not reported	Medical	Bespoke	Item	Not reported	Item	Not reported						
Giarelli 2012^48^		34/37	Nursing	Bespoke	Raw	Not reported	Raw	Not reported						
Malow 2023^49^		37/44	Medical, Nursing	Bespoke										
				Cohort 1	20.1	2.7					21.0*	2.4		
				Cohort 2	18.9	2.0					20.6*	2.1		
Mazurek 2020^50^		132/148	Medical (General practice, Paediatrics), Nursing	Bespoke	56	Not reported			62.0***	Not reported				
Mazurek 2020^51^		12/16	Medical (General practice, Paediatrics), Nursing	Bespoke	67.3	11.5			71.9	12.6				
McGonigle 2014^52^		110/110	Nursing	Bespoke	3.7	Not reported	3.9**	Not reported						

Study identifier	Attitudes								
	Scale	Pre		Post		3 Months		12 Months	
		Mean	s.d.	Mean	s.d.	Mean	s.d.	Mean	s.d.
**Intellectual disability – clinical care of adults**									
Dagnan 2018^19^									
PWP	ATTID	8.3	2.0	9.4	1.7	9.2*	2.2		
HIP		8.1	2.0	9.1	1.8	8.9*	1.6		
Eagleson 2022^20^	Bespoke	8.51	Not reported			8.8*	Not reported	8.45	Not reported
Harper 1992^21^									
Melville 2006^22^									
Written									
Written + Face to face									
Control									
Bessell 2023^23^									
Intervention									
Control									
**Intellectual and developmental disabilities – clinical care of children**									
McConkey 2014^24^									
Sadoo 2022^25^									
**Autism – screening in children**									
Balogh 2015^26^ Intervention Controls									
Bordini 2015^27^									
Carbone 2016^28^									
Johnson 2012^29^									
Lucarelli 2018^30^	Bespoke	4.7	0.7			4.7	0.7		
Mahoney 2023^31^									
Mazurek 2018^32^									
Swanson 2014^33^									
van’t Hof 2021^34^	CAMI	Item	Not reported	Item	Not reported				
**Autism – clinical care of children**									
Ashburner 2015^35^									
Bellesheim 2020^36^									
Donnelly 2020^37^	EBPAS	64.4	11.8	73.2***	12.0				
Eray 2017^38^									
Silva 2018^39^	KAP	42.1	8.6	66.5	9.1				
Gore 2024^40^									
Jonsdottir 2020^41^									
Ong 2021^42^									
Pasco 2014^43^									
Sengupta 2022^44^									
**Autism – Clinical care of adults**									
Ben-Sasson 2018^45^									
Dreiling 2022^46^									
Giachetto 2019^47^									
Giarelli 2012^48^									
Malow 2023^49^									
Mazurek 2020^50^									
Mazurek 2020^51^									
McGonigle 2014^52^									

PWP, psychological well-being practitioner; HIP, high-intensity practitioner; ATTID, Attitudes to the Treatment of People with Intellectual Disabilities in Mainstream Services; CAMI, Dutch translation of the Community Attitudes to Mental Illness questionnaire; EBPAS, Evidence-Based Practice Attitude Scale; KAP, Knowledge, Attitudes, Practice; ASD KSEQ, Autism Spectrum Disorder Knowledge and Self-Efficacy Questionnaire.

a. Follow-up was at 18 months. Study identifier: only the first author is noted, with year of publication. Bespoke refers to unpublished measures, constructed for purpose by authors. Data points: 0 = immediately pre- and post- the intervention. *P < 0.05, **P < 0.01, ***P < 0.001.

**Table 7:** Confidence and self-efficacy quantitative results

Study identifier	Participants		Confidence								
	Completed	Profession	Scale	Pre		Post		3 months		6 months	
				Mean	s.d.	Mean	s.d.	Mean	s.d.	Mean	s.d.
**Intellectual disability – clinical care of adults**											
Dagnan 2018^19^	42/68	Allied health	TCS-ID								
PWP				29.8	8.6	41.4	7.0	40.1***	8.0		
HIP				21.3	8.7	32.1	6.4	29.9***	10.2		
Eagleson 2022^20^	60/351	Medical, Nursing, Allied health									
Harper 1992^21^	31/Not reported	Medical, Medical students, Nursing									
Melville 2006^22^	63/79	Nursing									
Written											
Written + Face to face											
Control											
Bessell 2023^23^	62/101	Medical, Nursing, Allied health									
Intervention			Bespoke	2.7	0.5	3.5*	0.5				
Control				2.6	0.7	2.8	0.8				
**Intellectual and developmental disabilities – clinical care of children**											
McConkey 2014^24^	19/19	Nursing	Bespoke	21	Not reported	16	Not reported				
Sadoo 2022^25^	64/93	Medical, Nursing	Bespoke	2.7	Not reported					4.7***	Not reported
**Autism – screening in children**											
Balogh 2015^26^	26/Not reported	Medical, Nursing	Bespoke								
Intervention				21.3**	6.1					26.2***	6.0
Control				21.5	4.9					22.4	6.6
Bordini 2015^27^	22/29	Medical (Paediatrics, General practice)									
Carbone 2016^28^	43/43	Medical (General practice)									
Johnson 2012^29^	599/604	Nursing									
Lucarelli 2018^30^	54/129	Nursing, Allied health									
Mahoney 2023^31^	107/300	Nursing	Bespoke	3.7	0.8	4.0***	0.7				
Mazurek 2018^32^	18/26	Medical (General practice), Nursing									
Swanson 2014^33^	118/Not reported	Medical (Paediatrics)	Bespoke	Item		Item					
van’t Hof 2021^34^	78/93	Medical	Bespoke	29.0	5.6	37.7**	2.8	37.0**	4.1		
**Autism – clinical care of children**											
Ashburner 2015^35^	32/Not reported	Allied health (Occupational therapy)	Bespoke	61.9	17.8	77.1***	9.4				
Bellesheim 2020^36^	28/Not reported	Medical									
Donnelly 2020^37^	233/308	Medical (Paediatrics), Nursing, Allied health	CCPAI	17.0	4.2	19.2***	1.1				
Eray 2017^38^	75/79	Medical (General practice)									
Silva 2018^39^	14/14	Nursing, Allied health									
Gore 2024^40^	38/344	Nursing									
Jonsdottir 2020^41^	56/56	Medical, Nursing	Bespoke	2.1	0.5	3.1***	0.3				
Ong 2021^42^	8/12	Allied health	Bespoke	3.3	1.1	4.0	0.6				
Pasco 2014^43^	329/588	Medical, Nursing, Allied health									
Sengupta 2022^44^	62/88	Medical (Paediatrics)									
**Autism – clinical care of adults**											
Ben-Sasson 2018^45^	26/Not reported	Allied health (Physiotherapy)									
Dreiling 2022^46^	51/86	Allied health									
Giachetto 2019^47^	38/Not reported	Medical									
Giarelli 2012^48^	34/37	Nursing									
Malow 2023^48^	37/44	Medical, Nursing									
Mazurek 2020^50^	132/148	Medical (Paediatrics, General practice), Nursing									
Mazurek 2020^51^	12/16	Medical (Paediatrics, General practice), Nursing									
McGonigle 2014^52^	110/110	Nursing	Bespoke	3.0	Not reported	3.8	Not reported				

Study identifier	Self-efficacy										
	Scale	Pre		Post		3 months		6 months		12 months	
		Mean	s.d.	Mean	s.d.	Mean	s.d.	Mean	s.d.	Mean	s.d.
**Intellectual disability – clinical care of adults**											
Dagnan 2018^19^	GSE										
PWP		8.3	2.0	9.4	1,7	9.2*	2.2				
HIP		8.1	2.0	9.1	1.8	8.9*	1.6				
Eagleson 2022^20^	Bespoke	8.5	Not reported			8.8*	Not reported			8.45	Not reported
Harper 1992^21^											
Melville 2006^22^	Bespoke										
Written		8.9	2.1			9.2	2.1				
Written + Face to face		9.2	2.1			8.9	2.5				
Control		8.8	2.4			8.3	2.0				
Bessell 2023^23^											
Intervention											
Control											
**Intellectual and developmental disabilities – clinical care of children**											
McConkey 2014^24^											
Sadoo 2022^25^											
**Autism – screening in children**											
Balogh 2015^26^											
Intervention											
Control											
Bordini 2015^27^											
Carbone 2016^28^	Bespoke	Item	Not reported	Item	Not reported						
Johnson 2012^29^											
Lucarelli 2018^30^											
Mahoney 2023^31^	Bespoke	3.5	0.8	3.7***	0.7						
Mazurek 2018^32^	PCASE	201.4	44.3							269.3***	31.8
Swanson 2014^33^											
van’t Hof 2021^34^											
**Autism – clinical care of children**											
Ashburner 2015^35^											
Bellesheim 2020^36^											
Donnelly 2020^37^											
Eray 2017^38^											
Silva 2018^39^											
Gore 2024^40^	Bespoke	4.0	0.6	4.4**	0.7						
Jonsdottir 2020^41^											
Ong 2021^42^											
Pasco 2014^43^											
Sengupta 2022^44^	PCASE	2.7	Not reported	3.5***	Not reported						
**Autism – clinical care of adults**											
Ben-Sasson 2018^45^	Bespoke	2.0	0.6	2.4*	0.6						
Dreiling 2022^46^	PCASE	65.0	13.4					85.3***	2.6		
Giachetto 2019^47^											
Giarelli 2012^48^											
Malow 2023^49^	Bespoke										
Cohort 1		18.9	2.7					20.3*	2.4		
Cohort 2		18.9	2.0					20.6*	2.1		
Mazurek 2020^50^	Bespoke	42.0	Not reported			61.0***	Not reported				
Mazurek 2020^51^	PCASE	181.9	54.0			210.5*	38.9				
McGonigle 2014^52^											

TCS-ID, Therapy Confidence Scale–Intellectual Disabilities; GSE, General Self-Efficacy Scale; PCASE, Adapted, shortened version of the Primary Care Autism Self-Efficacy survey; KAP, Knowledge, Attitudes, Practice; AKQ-P, Autism Spectrum Disorder Knowledge Questionnaire – Physician edition; CAMI, Dutch translation of the Community Attitudes to Mental Illness questionnaire; CCPAI, Confidence in Coping with Patient Aggression Instrument. Study identifier: only the first author is noted, with year of publication. Bespoke refers to unpublished measures, constructed for purpose by authors. Data points: 0 = immediately pre- and post- the intervention. *P < 0.05, **P < 0.01, ***P < 0.001.

**Table 8:** Behaviour results

Study identifier	Follow-up duration	Measure		Score			
				Pre-education		Post-education	
				Mean	s.d.	Mean	s.d.
Balogh 2015^26^	6 months	Self-reported use of guidelines	Control	7.7	4.2	7.8	3.4
			Intervention	7.8	3.2	12.8***	3.5
		Self-reported periodic health examinations	Control	31.8	10.5	32.5	10.9
			Intervention	34.9	7.2	37.2	10.9
		Self-reported assessment of behaviour change	Control	24.6	5.7	23.6	5.8
			Intervention	24.2	6.0	27.5	7.1
Bordini 2015^27^	4 months	Number of cases referred for autism diagnostic assessment/total cases		1/274	Not reported	13/229	Not reported
Carbone 2016^28^	4 years	ASD screening rates		15%	Not reported	91%***	Not reported
Mazurek 2018^32^	Immediately post-education completion	Administration of autism screening tool		80%	Not reported	100%*	Not reported
		Percentage of well-child visits in past 12 months that included an autism screen		65%	Not reported	91%*	Not reported
Swanson 2014^33^	Range: 0.75-3.5 years	Utilise more than one formal ASD screening measure		91%	Not reported	95%	Not reported
	Mean 1.54 years	Conduct independent ASD assessment		23%	Not reported	68%	Not reported
Bellesheim 2023^36^	12 months	Performance of general developmental screen		53.3%	Not reported	96.7%	Not reported
		Performance of M-CHAT ASD screening		68.3%	Not reported	97.1%	Not reported

M-CHAT, Modified Checklist for Autism in Toddlers; ASD, autism spectrum disorder. Study identifier: only the first author is noted, with year of publication. Data points: 0 = immediately pre- and post- the intervention. *P < 0.05, **P < 0.01, ***P < 0.001.

**Table 9:** Combined design characteristics of included studies

Category	Variable	Number
**Education focus**	Intellectual disability clinical care in adults	4
	Intellectual and developmental disabilities clinical care in children	3
	Autism diagnosis in children	9
	Autism clinical care in children	10
	Autism clinical care in adults	8
**Intervention**		
**Setting**		
High-income country	USA	15
	UK	2
	Australia	6
	Canada	1
	Israel	1
	Brazil	2
	Iceland	1
	The Netherlands	1
Low- and middle-income country	India	1
	Macedonia	1
	Uganda	1
	Uruguay	1
	Turkey	1
	Romania	1
**Methods**		
Consumer involvement	Co-design only	2
	Co-delivery only	5
	Co-design and co-delivery	1
Mode of delivery	Online only	13
	Face to face only	12
	Face to face and online	7
	Written and video	1
	Written and face to face	1
Format	Interactive	9
	Didactic	12
	ECHO	7
	Mixed	5
	Other (co-mentoring)	1
Duration	<1 h	1
	1**–**5 h	9
	6**–**10 h	4
	11**–**20 h	12
	>20 h	3
	Not stated	5
Setting	Primary care	8
	Community health	19
	Paediatric hospital	1
	General hospital	6
	Mental health in-patient	2
	Community mental health	3
	Emergency department	2
	Broad healthcare setting	2
Design	Single-group pre-test/post-test	30
	Retrospective single-group post-test	1
	Waitlist control	1
	Partial stepped wedge randomised controlled trial	1
	Non-randomised two group	1
	Mixed quantitative + qualitative	5
	Qualitative only	0
Domains measured	Knowledge	28
	Attitude	7
	Confidence	13
	Self-efficacy	11
	Skills	2
	Behaviours	6
	Satisfaction	4
Measures	Studies using published validated measures	9
	Studies using bespoke measures only	25
Data time points	Pre- and immediate-post only	15
	Pre- and immediate-post and up to 3 months	8
	Pre- and immediate-post and >3 to 6 months	3
	Pre- and immediate-post and >6 to 12 months	4
	Pre- and immediate-post and >12 months	2

Table 10 describes the validated scales used in these studies in further detail. The majority (25 out of 34) of studies used bespoke measures that were designed for purpose and not published or validated. Of the nine studies that used outcome measures that were published and validated, ten different scales were used, and none stood out as more commonly used. Some scales were developed specifically for use in this area, whereas others were not. The ASD Knowledge and Self-Efficacy Questionnaire^53^ and Autism Spectrum Disorder Knowledge Questionnaire – Physician’s Edition (AKQ-P),^54^ were designed to measure knowledge of autism. By contrast, only one scale measuring attitudes was specifically designed for use in relation to intellectual disability or autism, the Attitudes Towards Intellectual Disability (ATTID) scale,^55^ whereas the other two published scales were designed for assessing attitudes to evidence-based practice (Evidence-Based Practice Attitude Scale, EBPAS)^56^ or mental illness (Community Attitudes Towards the Mentally Ill, CAMI).^57^ Confidence was measured using the targeted Therapy Confidence Scale – Intellectual Disabilities (TCS-ID)^58^ and the non-disability specific Confidence in Coping with Patient Aggression Instrument.^59^ Self-efficacy scales were specific for intellectual disability (General Self-Efficacy Scale, GSE)^60^ or autism (Primary Care Autism Self-Efficacy survey, PCASE).^61^ The Challenging Behaviour Attributions Scale (CHABA) was used to assess beliefs around aggression.^62^

**Table 10:** Outcome measures

Scale name	Scale focus	Scale author and year	Scale design	Study using this scale
**Intellectual disability**				
Attitudes Towards Intellectual Disability (ATTID)	Attitudes	Morin 2013^55^	5 items extracted from larger 67 item scale	Dagnan 2018^19^
Therapy Confidence Scale – IntellectualDisabilities (TCS-ID)	Confidence	Dagnan 2015^58^	14 items, 5 point Likert	Dagnan 2018^19^
General Self-Efficacy Scale (GSE)	Self-efficacy	Dagnan 2015^58^	5 items	Dagnan 2018^19^
**Autism spectrum disorder**				
ASD Knowledge and Self-Efficacy Questionnaire	Knowledge, self-efficacy	Atun-Einy 2018^53^	79 items	Ben-Sasson 2018^45^
Evidence-Based Practice Attitude Scale (EBPAS)	Attitude	Aarons 2010^56^	15 items, 5 point Likert	Donnelly 2020^37^
Confidence in Coping with Patient Aggression Instrument	Confidence	Thackrey 1987^59^	10 items, Likert scale	Donnelly 2020^37^
Challenging Behaviour Attributions Scale (CHABA)	Aggression	Hastings 2007^62^	39 items, 5 point Likert scale	Donnelly 2020^37^
Primary Care Autism Self-Efficacy survey (PCASE)	Self-efficacy	Mazurek 2016^61^	57 items, 6 point Likert	Mazurek 2019^32^ Mazurek 2020^51^ Dreiling 2022^a^ ^46^ Sengupta 2022^a^ ^44^
Autism Spectrum Disorder Knowledge Questionnaire – Physician’s Edition (AKQ-P)	Knowledge	van’t Hof 2020^54^	32 multiple choice questions	van’t Hof 2021^34^
Community Attitudes Towards the Mentally Ill (CAMI)	Attitudes	Taylor 1981^57^	40 items, 5 point Likert	van’t Hof 2021^34^

a. abbreviated form. Scale author and year, Study using this scale: only first author of publication is noted, with year of publication.

Table 11 demonstrates knowledge and confidence had the most statistically significant improvements, although fewer studies measured outcomes other than knowledge. Qualitative findings revealed various knowledge and skills gains across interventions. One study discussed that although participants thought they already knew about autism, the intervention helped them ‘see that there was much more to learn’.^44^ An intervention that focused on therapists working with people intellectual disability reported increased awareness of the needs of people with intellectual disability and how they could improve their practice.^19^ Examples of changes that were identified included adaptation of materials, nature of communication and the interventions used in therapy.^19,25^ Changes in attitudes were also highlighted, and related to the involvement of a parent of an autistic child as part of the expert hub providing education, which gave greater awareness and insights into the experience of the autistic child and their family.^44^ One paper evaluated the use of co-mentoring as a technique that was chosen to improve the uptake of learnings in the workplace following a didactic training course. Benefits included the opportunity to discuss learnings and how they might be implemented, challenges were the logistic challenges in a busy workplace.^35^

**Table 11:** Synthesis of quantitative findings

Variable	Statistical significance	Number
Improved knowledge	*P* < 0.001	10
	*P* < 0.01	5
	*P* < 0.05	6
	Not significant	4
	No analysis reported	3
	Total	28
Improved attitudes	*P* < 0.001	2
	*P* < 0.01	1
	*P* < 0.05	2
	Not significant	2
	Total	7
Improved confidence	*P* < 0.001	7
	*P* < 0.01	1
	*P* < 0.05	1
	No analysis reported	2
	Not significant	2
	Total	13
Improved self-efficacy	*P* < 0.001	5
	*P* < 0.01	1
	*P* < 0.05	3
	Not reported	1
	Not significant	1
	Total	11

### Reporting biases

Few studies performed statistical analyses on collected data or analysed only single questionnaire items. Although no results were missing, reporting bias likely exists because all studies with statistical analysis reported at least one positive finding, and it would be expected that there would be some studies in this area that showed no change in measured parameters.

## Discussion

This systematic review supports education’s effectiveness in improving healthcare professionals’ knowledge, attitudes, confidence and self-efficacy when working with individuals with intellectual or developmental disabilities. All 29 purely quantitative studies showed significant improvements in at least one measured aspect (Table 10), with five mixed-methods studies providing additional qualitative support for effectiveness and implementation (Table 4). This review’s focus on healthcare professionals means its findings have applicability in the workplace, where issues such as the cost and motivation for employees to participate in education differ from those relevant to undergraduate training. However, significant limitations weaken the quantitative findings, including selection bias, sample sizes, non-published and non-validated outcome measures, and the absence of negative findings across all studies. Only nine out of 34 studies used validated measures (with four using the same instrument) (Table 10), only five studies used objective outcome measures (Table 8) and some qualitative studies lacked robust coding and analysis methods. Together, these limitations weaken the conclusions that can be drawn from this systematic review.

There is an established international lack of health professional knowledge and confidence in this area.^2,3,63,64^ The positive findings in both high- and low- and middle-income countries pomote the importance of prioritising education for health professionals and students. The review further demonstrates that education has been successfully delivered to improve screening and diagnosis in children, as well as clinical care for all age groups, across intellectual and developmental disabilities, including autism. However, most papers (22 out of 34) focused on improving diagnosis and clinical care in children, with the vast majority (19 out of 22) specifically addressing autism. This distribution may reflect the relatively recent increased awareness and educational needs regarding autism in children, with these studies potentially employing more rigorous methods because they are more recent. Notably, there appears to be a relative lack of evaluated educational interventions focused on improving clinical care for adults with intellectual or developmental disabilities, including those on the autism spectrum.

### Improving knowledge, attitudes, confidence and self-efficacy

Recent calls for improved health professional education evaluation^16^ prompted development of the Standards for Quality Improvement Reporting Excellence in Education (SQUIRE-EDU)^65^ and measures to assess quality of medical education research.^11,16^ The MERSQI measure, used to assess quality in this review, ranks outcomes in increasing order of quality, with lowest ranking scores for subjective self-report measures of satisfaction, attitudes, perceptions and general facts, followed by knowledge and skills, followed by behaviours; and highest ranking scores for the use of patient/healthcare outcomes. Most studies in this systematic review scored the lowest scores. There was some measurement of behaviours in six studies (Table 8), such as health professionals performing periodic health assessments, or blinded patient assessments of skills to assess diagnostic accuracy in one study (Tables 2 and 3).^20^ The challenge in the area of intellectual and developmental disabilities is that, apart from screening and diagnosis in children, there are few behavioural or patient outcome parameters that can be measured in adults. One potential objective measure could be the number of patients identified with intellectual disability in records pre- and post-intervention.

This review’s synthesis of qualitative and quantitative results suggests that education is especially effective in improving knowledge in this area. However, it is of concern that attitudes of health professionals, a major source for concern in this area, was relatively under-investigated quantitatively, reported in only seven papers. This is despite the availability of several published and validated scales to measure attitudes in intellectual disability,^66–68^ and some in autism.^69,70^ It is also noteworthy that the synthesis of quantitative knowledge outcomes was problematic, as most scales were unpublished and situation specific. It was not clear what type of knowledge was improved in most of the quantitative results, and how generalisable that might be to healthcare for people with intellectual disability and those on the autism spectrum. Qualitative findings did suggest improvements in knowledge and provided some examples of the ways that this knowledge improved practice. Confidence, and the closely related concept of self-efficacy, were measured, with confidence showing particularly impressive improvements in eight of the ten studies that measured it.

### Co-design and delivery

The value of involving consumers in research has gained increasing recognition, with supporting policies and resources emerging in countries such as the UK, Canada and Australia.^71–73^ Inclusion of individuals with lived experience in the design and delivery of intellectual and developmental disabilities health education and research is now widely acknowledged as a priority.^74^ However, this review found such involvement to be rare – only one study reported both co-design and co-delivery. Although seven different studies involved people with lived experience in the delivery of the education, only one reported feedback from participants on how this affected their educational experience. There can be challenges involving individuals with intellectual disability or those on the autism spectrum, but it is not a new practice. As early as 2008, Tracy and Iacono included tutors with intellectual disability in teaching medical students on communication with people with intellectual disability.^75^ This approach requires careful planning and support, and should be embedded early in project development, and considered in the evaluation of the education.

### Effective dosing and mode of delivery

This review also sought to examine whether there was a minimum dose of education necessary to improve outcomes. Education of paid employees is a financial burden to the employer, so this finding could help encourage efficient use of educational interventions in this area. In this review, approximately a third of interventions were 5 h or less in duration, and about a third were over 11 h. Unfortunately, the variety of measures used limits the strength of conclusions that can be drawn.

Guidance on the most effective modality can be considered by the finding of generally positive results across all modes of delivery, including the 13 out of 34 studies that used online delivery only. This shows promise for the effectiveness of online education, which has greater flexibility in remote areas, and in ease of access and administration. However, further research is required to allow more robust consideration of these variables.

### Limitations

One of the main limitations of this review is its focus on restricting inclusion of papers that described either qualitative or quantitative outcome measures. This was necessary to ask the key question in relation to effectiveness of education. However, it means that the findings relate only to this group of evaluated educational interventions, and these may not be representative of all educational interventions in this area. The requirement for published outcomes also means that any papers with narratively discussed outcomes would also have been excluded. The heterogeneous focus of included studies (e.g. diagnosis versus clinical care) may also have made comparisons between education with different types of approaches less reliable or valid.

### Implications and future directions

This systematic review found that educational interventions consistently improved healthcare professionals’ knowledge, attitudes, self-efficacy, confidence and skills. However, many studies lacked validated or comparable outcome measures, limiting the strength of findings. Co-design and co-delivery were uncommon and should be prioritised in future research and incorporated into evaluation. There is a clear need for a validated knowledge scale tailored to health professionals to support comparisons between studies. Qualitative components provided valuable insights into intervention impact, but overall, more robust outcome measures are needed to strengthen future study designs.

In conclusion, this mixed-methods systematic review found that educational interventions had positive effects on health professionals’ knowledge, skills, confidence and or self-efficacy. Health professional participants often expressed surprise at how much there was to learn, and reported that training improved the quality of the care they provided to people with intellectual disability and those on the autism spectrum. Involving people with intellectual disability and those on the autism spectrum in the design and delivery of interventions was noticeably scarce, and should be prioritised in future efforts. Further research is needed to determine the most effective delivery modes and duration of education.

### Lived experience summary (by R.d.G. and C.F.)

Education is important for health professionals to help them give better care to people with intellectual disability and autistic people. Some need to learn more about how to communicate with people with intellectual disability and autistic people, and how to listen better. Otherwise, people with disabilities don’t get the care they need.

It would be good for this education to be mandatory everywhere, like it is in England, because then all health staff would learn more about people with disabilities and how to care for them better.

It’s important that people with disabilities are involved in the design, so that they can say what people with disabilities want health professionals to hear, so they are teaching the right things. Involving people with disability in giving the education is a good way to show health professionals what it’s like to have a disability, so that they can understand better and be more familiar with disability.

Measuring if the education works is important because people with a disability need to be able to see health professionals who can offer equal service to people with a disability to the general population. It’s important that health professionals learn from the education and that it can be shown that they have learnt, to offer the best healthcare to people with a disability.

## Supporting information

Franklin et al. supplementary material Franklin et al. supplementary material

## Data Availability

The data-sets produced during the current study will be available on reasonable request from the corresponding author, C.F.

## Supplementary material

The supplementary material is available online at https://doi.org/10.1192/bjo.2025.10842
